# Supplementary material for: Structural Characterization of Glycerophosphorylated and Succinylated Cyclic β-(1→2)-d-Glucan Produced by Sinorhizobium mliloti 1021
Source: Polymers (Basel). 2020 Sep 12;12(9):2073. doi: 10.3390/polym12092073 (PMC7569799; doi:10.3390/polym12092073)
Supplement: Supplementary file 1 [file polymers-12-02073-s001.pdf]

polymers

MDPI

Supplementary Materials

Structural Characterization of Glycerophosphorylated and Succinylated Cyclic β-(1→2)-D-glucan Produced by *Sinorhizobium meliloti* 1021

Hyojeong Lee [1], Seonmok Kim [1], Yohan Kim [1] and Seunho Jung [1,2,*]

[1] Department of Bioscience and Biotechnology, Microbial Carbohydrate Resource Bank (MCRB), Center for Biotechnology Research in UBITA (CBRU), Konkuk University, Seoul 05029, Korea; lhjeong199@naver.com (H.L.); gkdurk9999@naver.com (S.K.); shsks1@hanmail.net (Y.K.)

[2] Department of Systems Biotechnology & Institute for Ubiquitous Information Technology and Applications (UBITA), Center for Biotechnology Research in UBITA (CBRU), Konkuk University, Seoul 05029, Korea

* Correspondence: shjung@konkuk.ac.kr; Tel.:+82-2-450-3520

Received: 28 August 2020; Accepted: 10 September 2020; Published: date

Table of Contents

1. ^{1}H NMR spectra of F2, F3 and F4 fraction ······ p2

2. ^{13}C NMR spectra of F2, F3 and F4 fraction ······ p3

3. HSQC NMR spectra of F2, F3 and F4 fraction ······ p5

4. ^{31}P NMR spectra of F2, F3 and F4 fraction ······ p7

(**a**)

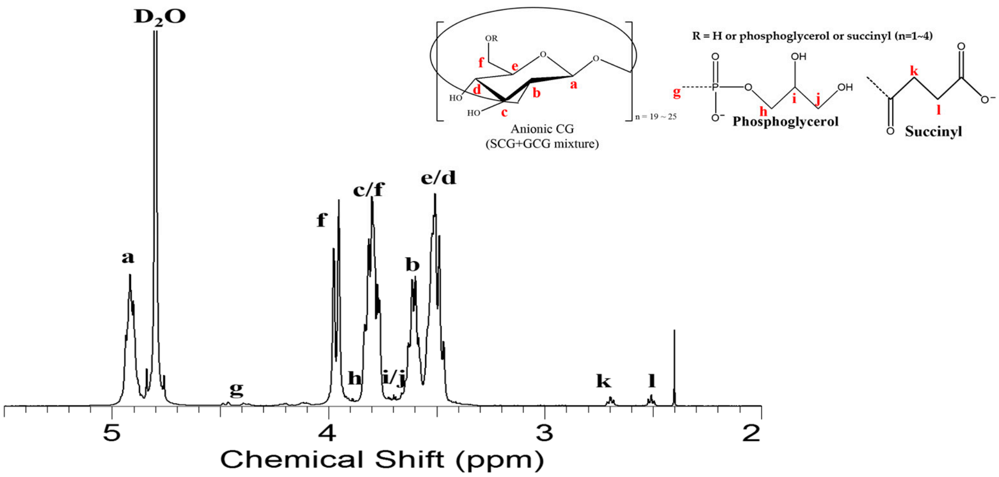

(**b**)

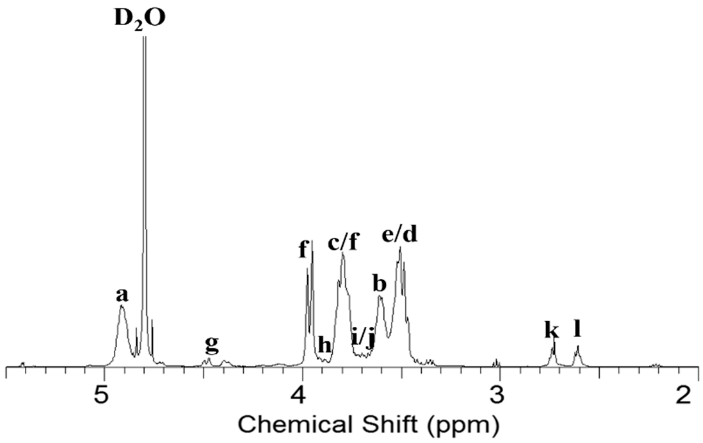

(**c**)

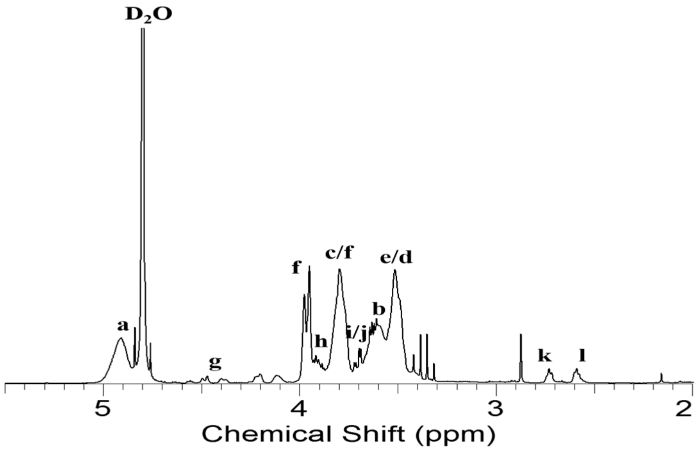

Figure S1. ^{1}H NMR spectra of (a) F2, (b) F3 and (c) F4 fraction

(**a**)

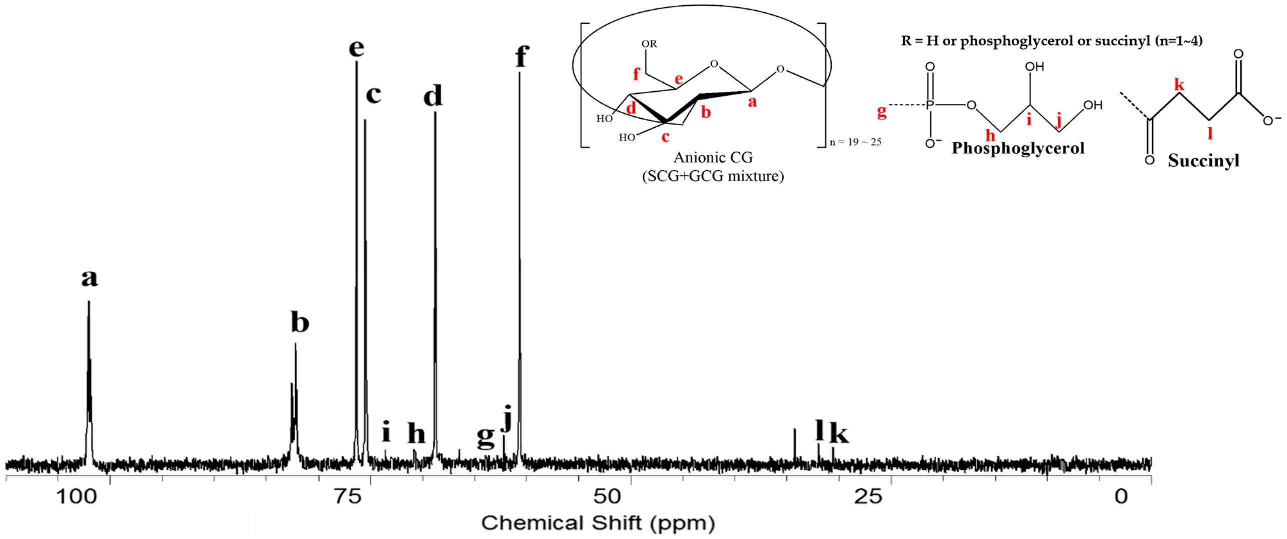
R = H or phosphoglycerol or succinyl (n=1~4)
Anionic CG
(SCG+GCG mixture)
n = 19 ~ 25
Phosphoglycerol
Succinyl
a
b
c
d
e
f
g
h
i
j
l
k
100
75
50
25
0
Chemical Shift (ppm)

(**b**)

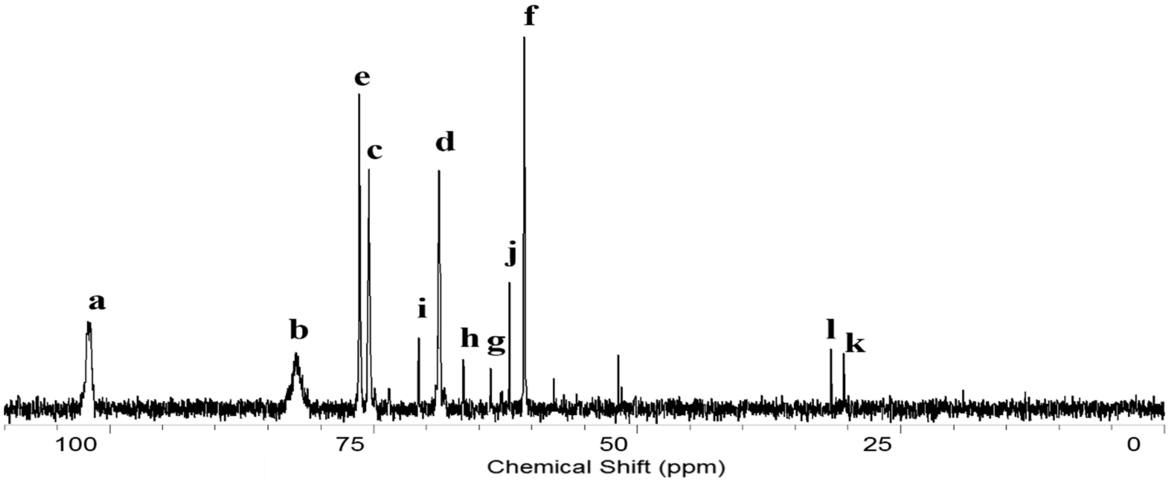
a
b
e
c
d
f
i
h
g
j
l
k
100
75
50
25
0
Chemical Shift (ppm)

(**c**)

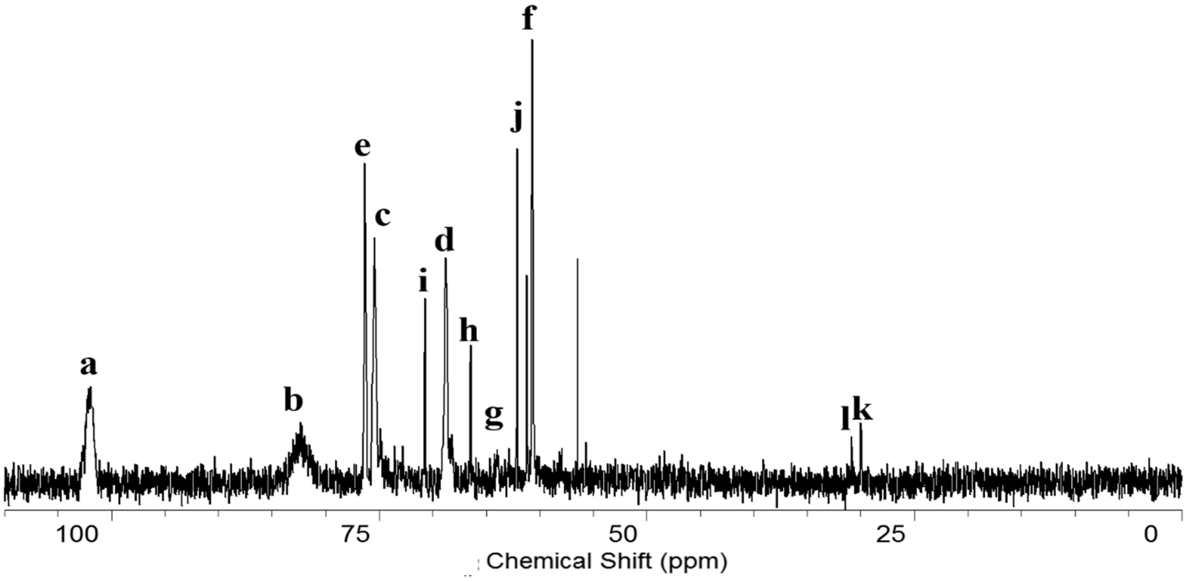

Figure S2. ^{13}C NMR spectra of (a) F2, (b) F3 and (c) F4 fraction.

(**a**)

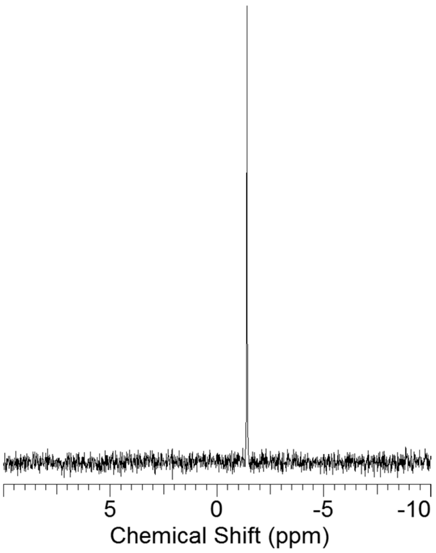

(**b**)

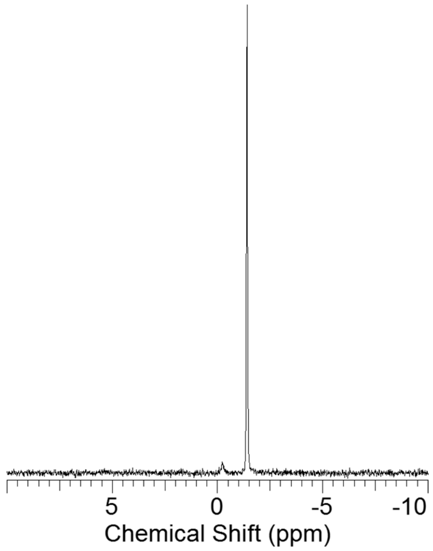

(**c**)

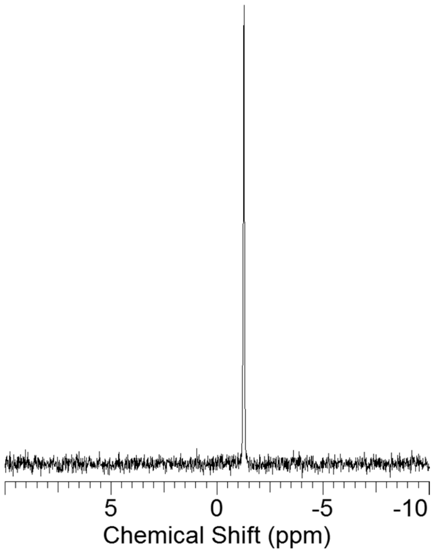

Figure S3. The proton-decoupled ^{31}P NMR spectra of (a) F2, (b) F3 and (c) F4 fraction.

(**a**)

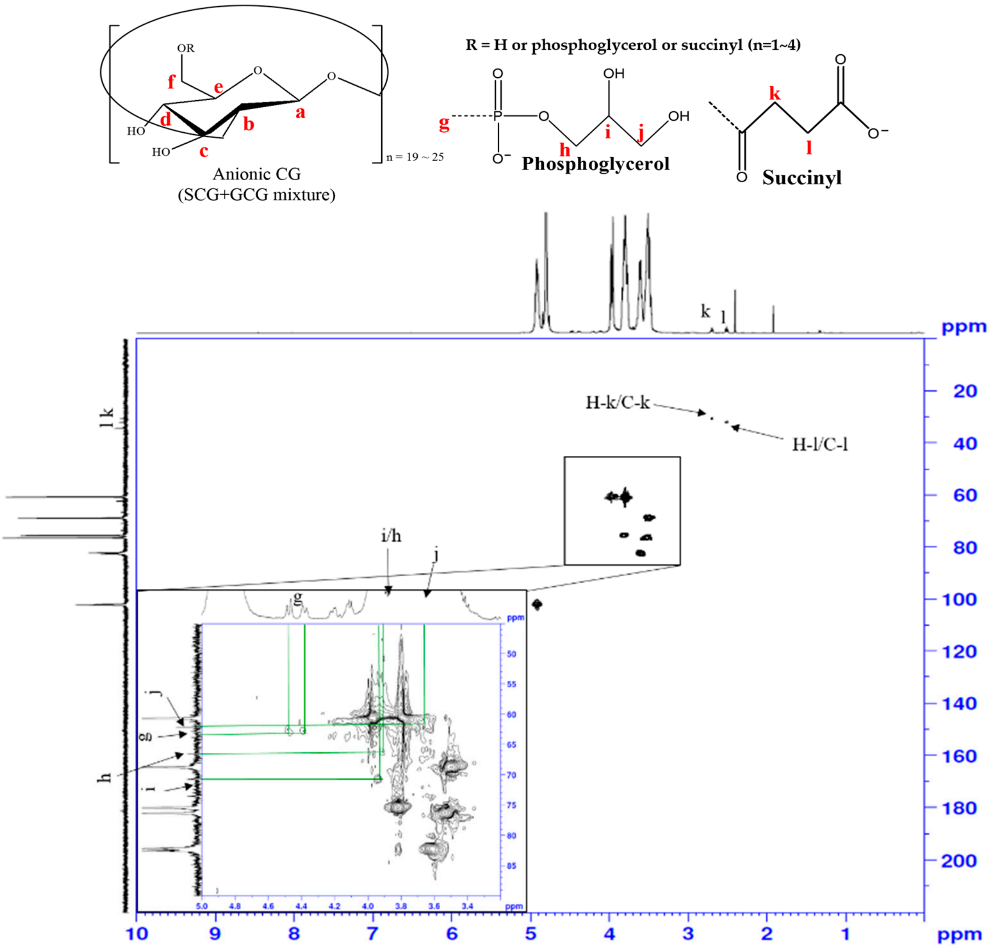
R = H or phosphoglycerol or succinyl (n=1~4)
Anionic CG
(SCG+GCG mixture)
n = 19 ~ 25
Phosphoglycerol
Succinyl
H-k/C-k
H-l/C-l
i/h
ppm

(**b**)

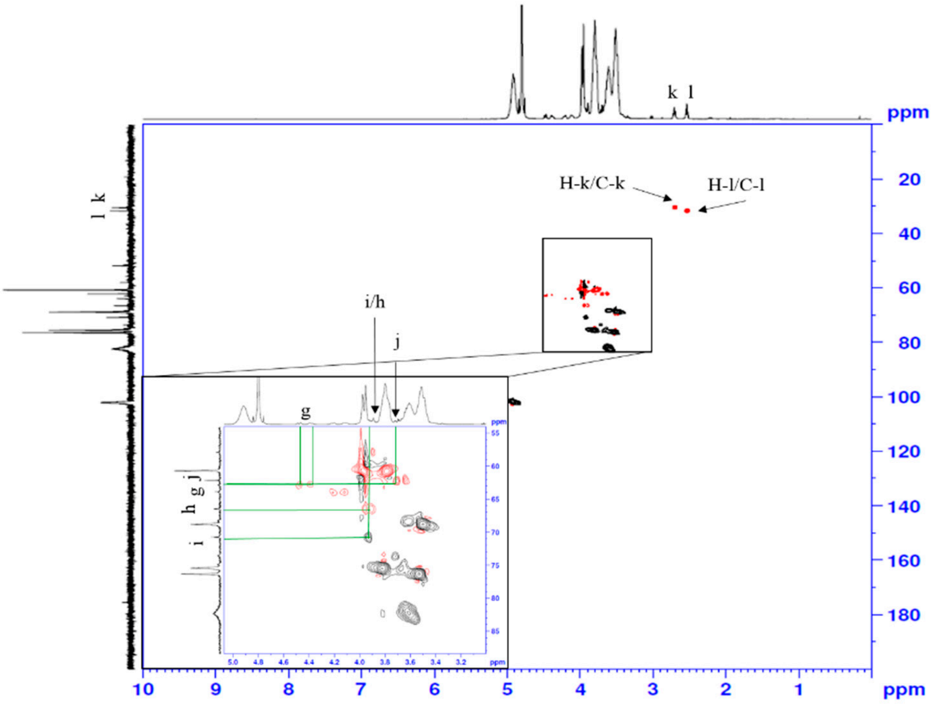

(**c**)

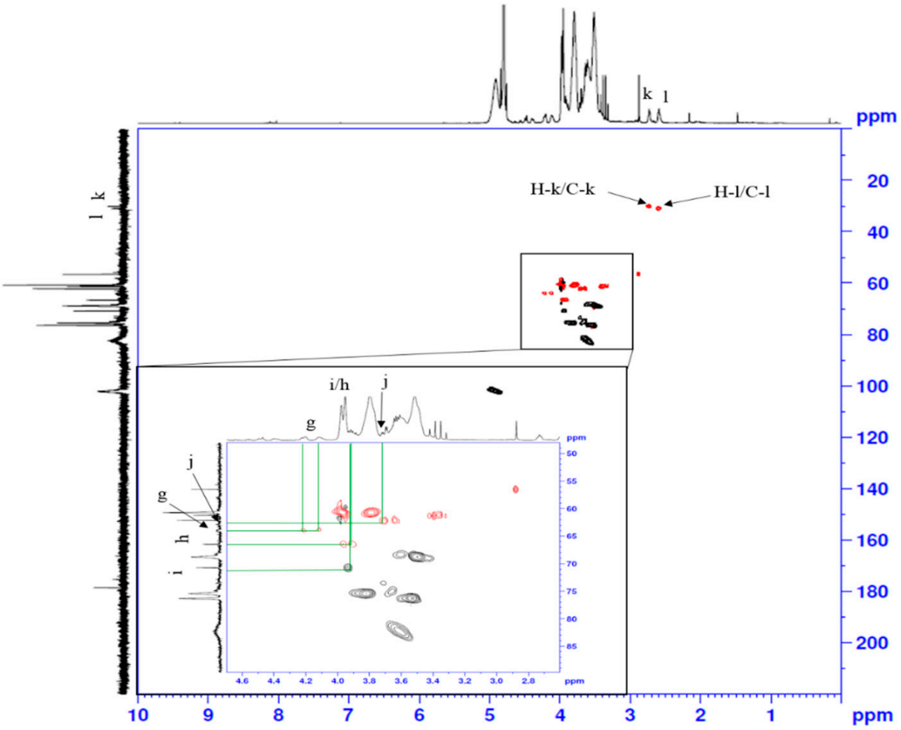

Figure S4. ^1H-^{13}C heteronuclear single quantum coherence (HSQC) NMR spectra of (a) F2, (b) F3 and (c) F4 fraction.
